# Recent Advances in Liquid Biopsy Based on Circulating Tumor DNA

**DOI:** 10.3390/jcm8111957

**Published:** 2019-11-13

**Authors:** Yu Sakuma, Keita Fujii, Jiayan Han, Ryou-u Takahashi

**Affiliations:** Department of Cellular and Molecular Biology, School of Pharmaceutical Sciences, Graduate School of Biomedical & Health Sciences, Hiroshima University, 1-2-3 Kasumi, Minami-ku, Hiroshima city, Hiroshima 734-8553, Japan; d183226@hiroshima-u.ac.jp (Y.S.); b163057@hiroshima-u.ac.jp (K.F.); d180761@hiroshima-u.ac.jp (J.H.)

Many types of cells secrete DNA, RNA, and proteins through microvesicles, such as exosomes and apoptotic bodies, for the purpose of extracellular communication. Therefore, in addition to circulating tumor cells (CTCs), secreted DNA, RNA, and protein in body fluids such as blood, urine, saliva, and milk are useful targets for liquid biopsy. Importantly, recent advances in technologies for the detection and characterization of CTCs and circulating tumor-derived factors have made liquid biopsy more rapid and sensitive than conventional tissue biopsy. 

Over the past decade, multiple studies have demonstrated that non-coding RNAs (ncRNAs) such as microRNAs (miRNAs) and long intergenic RNAs (lincRNAs) are important not only in cancer therapy [1], but also in cancer diagnosis: ncRNAs secreted from various types of cells through microvesicles [2] play critical roles in cancer biology, including the development of the tumor microenvironment and metastasis [3,4]. For example, in brain metastasis of breast cancer cells, secreted miRNAs disrupt the blood–brain barrier prior to cancer cell invasion and metastasis [4]. Therefore, from the standpoint of their clinical potential as therapeutic targets and diagnostic markers, secreted ncRNAs are attractive target molecules for liquid biopsy.

Recent progress in the technologies for detection and characterization of CTCs and circulating tumor DNAs (ctDNAs), including digital PCR, next-generation sequencing, and single-cell sorting coupled with PCR, has enabled more precise quantification of tumor-specific mutations in CTCs and ctDNAs, as well as more accurate identification and monitoring of minimal residual disease. Following the Food and Drug Administration approval of a ctDNA-based liquid biopsy test for lung cancer patients, technologies for liquid biopsy are being put into practical use for cancer diagnosis. In this review, Krishnamurthy et al. introduce and summarize recent advances in ctDNA analysis for cancer diagnosis [5]. They also discuss the remaining obstacles to fuller clinical utilization of ctDNA. This excellent review will give Journal of Clinical Medicine readers a better understanding of the current status of liquid biopsy and the prospects for the development of non-invasive and highly-sensitive biomarkers.
